# Supplementary material for: A parasite's perspective on data sharing
Source: Gigascience. 2018 Nov 5;7(11):giy129. doi: 10.1093/gigascience/giy129 (PMC6258825; doi:10.1093/gigascience/giy129)

GigaScience

# A Parasite's Perspective on Data Sharing

--Manuscript Draft--

| | |
|---|---|
| **Manuscript Number:** | GIGA-D-18-00388 |
| **Full Title:** | A Parasite's Perspective on Data Sharing |
| **Article Type:** | Commentary |
| **Funding Information:** | Gordon and Betty Moore Foundation (GBMF 4552) — Prof. Casey S. Greene<br>National Human Genome Research Institute (HG010067) — Prof. Casey S. Greene |
| **Abstract:** | Data generation is expensive in terms of both time and money. Sharing data enables the rapid replication, validation and application of discoveries, increasing the pace and accuracy of research. As research parasites, or users of other people's data, we recognize that a strong science ecosystem requires those who share best to be recognized. We find that widely accessible benchmark datasets have provided outsized benefits, and we hope that the benefits of sharing will also begin to accrue to individual investigators who share well. Funders can enhance progress by adjusting incentives to better support data sharers, which will make their programmatic investments more effective. We note some funders who are making such efforts. |
| **Corresponding Author:** | Casey S. Greene<br><br>UNITED STATES |
| **Corresponding Author Secondary Information:** | |
| **Corresponding Author's Institution:** | |
| **Corresponding Author's Secondary Institution:** | |
| **First Author:** | YoSon Park |
| **First Author Secondary Information:** | |
| **Order of Authors:** | YoSon Park<br>Casey S. Greene |
| **Order of Authors Secondary Information:** | |
| **Additional Information:** | |
| **Question** | **Response** |
| Are you submitting this manuscript to a special series or article collection? | No |
| **Experimental design and statistics**<br><br>Full details of the experimental design and statistical methods used should be given in the Methods section, as detailed in our Minimum Standards Reporting Checklist. Information essential to interpreting the data presented should be made available in the figure legends. | No |

Powered by Editorial Manager® and ProduXion Manager® from Aries Systems Corporation

| | |
|---|---|
| Have you included all the information requested in your manuscript? | |
| If not, please give reasons for any omissions below.<br><br>as follow-up to "**Experimental design and statistics**<br><br>Full details of the experimental design and statistical methods used should be given in the Methods section, as detailed in our Minimum Standards Reporting Checklist. Information essential to interpreting the data presented should be made available in the figure legends.<br><br>Have you included all the information requested in your manuscript?<br><br>" | This is a commentary. |
| **Resources**<br><br>A description of all resources used, including antibodies, cell lines, animals and software tools, with enough information to allow them to be uniquely identified, should be included in the Methods section. Authors are strongly encouraged to cite Research Resource Identifiers (RRIDs) for antibodies, model organisms and tools, where possible.<br><br>Have you included the information requested as detailed in our Minimum Standards Reporting Checklist? | No |
| If not, please give reasons for any omissions below.<br><br>as follow-up to "**Resources**<br><br>A description of all resources used, | This is a commentary. |

| | |
|---|---|
| including antibodies, cell lines, animals and software tools, with enough information to allow them to be uniquely identified, should be included in the Methods section. Authors are strongly encouraged to cite Research Resource Identifiers (RRIDs) for antibodies, model organisms and tools, where possible.<br><br>Have you included the information requested as detailed in our Minimum Standards Reporting Checklist?<br><br>" | |
| **Availability of data and materials**<br><br>All datasets and code on which the conclusions of the paper rely must be either included in your submission or deposited in publicly available repositories (where available and ethically appropriate), referencing such data using a unique identifier in the references and in the "Availability of Data and Materials" section of your manuscript.<br><br>Have you have met the above requirement as detailed in our Minimum Standards Reporting Checklist? | No |
| If not, please give reasons for any omissions below.<br><br>as follow-up to "**Availability of data and materials**<br><br>All datasets and code on which the conclusions of the paper rely must be either included in your submission or deposited in publicly available repositories (where available and ethically appropriate), referencing such data using a unique identifier in the references and in the "Availability of Data and Materials" section of your manuscript. | This is a commentary. |

| | |
|---|---|
| Have you have met the above requirement as detailed in our Minimum Standards Reporting Checklist?<br><br>" | |

Powered by Editorial Manager® and ProduXion Manager® from Aries Systems Corporation

Manuscript final version
Click here to access/download;Manuscript;2018_resource_sharing_commenta
Click here to view linked References

# A Parasite's Perspective on Data Sharing

YoSon Park[1] and Casey S. Greene[1,2]

ORCIDs:
YoSon Park ( 0000-0002-0465-4744 )
Casey S. Greene ( 0000-0001-8713-9213 )

[1] Department of Systems Pharmacology and Translational Therapeutics, Perelman School of Medicine University of Pennsylvania, Philadelphia, PA 19104
[2] Childhood Cancer Data Lab, Alex's Lemonade Stand Foundation, Philadelphia PA 19103

Corresponding author: Casey S Greene. Email: csgreene@mail.med.upenn.edu

**Abstract:**

Data generation is expensive in terms of both time and money. Sharing data enables the rapid replication, validation and application of discoveries, increasing the pace and accuracy of research. As research parasites, or users of other people's data, we recognize that a strong science ecosystem requires those who share best to be recognized. We find that widely accessible benchmark datasets have provided outsized benefits, and we hope that the benefits of sharing will also begin to accrue to individual investigators who share well. Funders can enhance progress by adjusting incentives to better support data sharers, which will make their programmatic investments more effective. We note some funders who are making such efforts.

**Keywords:** Data sharing, open data, research parasite.

**Main Text:**

The primary goal of scientific publishing is to disseminate findings to the community and disclose supporting observations that allow the community to infer their validity and robustness. Widely used forms of data release, such as making data available only upon reasonable requests, can delay sharing indefinitely. Though these practices reduce the scientists' ability to critically evaluate or build upon previous work, some journals still allow them. Some editors have questioned whether data sharing may bring harm to certain fields, going so far as to call someone who has re-analyzed shared data without providing co-authorships to sharers "research parasites" (1). This has given rise to "Research Parasite Awards" focused on data-reuse (2) and "Research Symbiont Awards" focused on data-sharing (3) that seek to recognize exemplar participants in this scientific ecosystem. As research parasites, we provide our perspective on some of the best examples of data sharing. We note how these practices provide an efficient path to accurate, impactful findings. We also discuss how funders can encourage innovation and improvements in science by preferring to support researchers who are sharing.

During the outbreak of the deadly Zika virus (ZIKV) in South and Central America in 2013 and 2014, the virus spread so rapidly that researchers studying it felt the need to share their data before completing the publication process. Acknowledging this, several researchers announced a data release, including sequence data of candidate strains, to the World Health Organization (WHO) to enable other researchers to develop vaccines (4). The Zika experimental-science team (ZEST), studying rhesus macaques infected with ZIKV, made their data public rather than publishing them in a journal, and continued to update their results daily for others to benefit from their progress (5). These scientists committed to sharing information rapidly during public health emergencies, but there were no guidelines for formal recognition and rewards for such efforts at the time.

There are also sharing-first projects outside of infectious disease research. As part of a £62 million initiative, the UK Biobank recruited 500,000 individuals from the UK aged between 40-69 in 2006-2010 and publicly released all generated data for *bona fide* research use (6). The consortium behind this effort collected and continues to collect extensive phenotypic data including various physical measurements, accelerometry, questionnaires, biochemical assays and genotyping data from all of its participants. The conventional model for novel, multi-site data collection is to restrict access to only a small set of participants who produce publications and other accomplishments linked to the resource. However, the UK Biobank is transforming the way public genetic and health data should be shared. This consortium, while providing strategically de-identified electronic health records, genotypes and other research data to benefit public health, explicitly stated that no resource users will be expected to add the UK Biobank as co-authors on their publications or share income generated from research using these resources.

Certain data resources undergo continuous updates; in an example from ecology, researchers are actively building platforms to embrace evolving or dynamic data (7). Yenni and colleagues describe an automated workflow where researchers can automatically integrate new data with versioning and archiving, and update associated manuscripts and expand relevant supplementary tables. This framework not only allows faster integration and automated analysis of continuously updated data, but also ensures systematic review of the workflow, faster dissemination of results and provides transparency for others to validate the study and its findings. Figure 1 depicts citations for publicly shared data between 2008 till present, and for the past decade, we estimated that approximately 7,324 publications benefited from these public resources.

If data sharing plays such a key role in more rapid and accurate scientific progress, how can this behavior be better encouraged? Funders have a unique role in the scientific ecosystem. Those working at universities and other research institutions often face incentive structures that are challenging for those who seek to make change, such as promotion and tenure committees that are not accustomed to crediting data sharing. Alberts et al. (8), in an article describing flaws in the biomedical research ecosystem, note that at many institutions a faculty salary is almost entirely covered on research grants. This increases universities' reliance on funder dollars and provides funders with an expanded role in driving evaluation metrics throughout the ecosystem.

Funders benefit when scientists find errors earlier and make discoveries sooner, and some are strengthening their resource sharing expectations. The Chan-Zuckerberg Initiative expects grantees to share protocols and preprints for projects that they fund (9). Alex's Lemonade Stand Foundation asks applicants to describe their past sharing behavior alongside their plans for sharing resources that result from the proposal, and both are assessed for impact (10). Since 2017, the Bill & Melinda Gates Foundation requires peer-reviewed publications and associated data to be discoverable and accessible openly and offers to pay for open access as needed. These are important changes because researchers who share face challenges due to the scientific norms around credit. These challenges include the costs associated with open access publishing and high-quality data sharing as well as the risks that errors could be found in their work or that others could extend their work causing them to lose future accomplishments and credit. By providing incentives for sharing, funders help to mitigate these perceived risks and thus improve the value of their investment. Funders can make their investments go further by supporting a culture among investigators that values behaviors that accelerate scientific progress.

## Note from the Editors

The 2019 Research Parasite Awards will be awarded at the 2019 Pacific Symposium on Biocomputing (PSB) in early January, and *GigaScience* for the third year running, is proud to support the Junior Parasite prize with a travel grant.

## Abbreviations

WHO, World Health Organisation; ZEST, Zika experimental-science team; ZIKV, Zika Virus.

## Competing Interests

The authors declare that they have no competing interests.


## Funding

This work is funded in part by grants from the Gordon and Betty Moore Foundation (GBMF 4552), Alex's Lemonade Stand Foundation (CCDL), and by 2018-182718 from the Chan Zuckerberg Initiative Donor-Advised Fund (DAF), an advised fund of the Silicon Valley Community Foundation to CSG. CSG and YP are funded by NIH R01 HG010067.


## Authors' contribution

| | |
|---|---|
| Writing – Original Draft Preparation | CSG and YP |
| Writing – Review and Editing | CSG and YP |
| Investigation | YP |


## Acknowledgements

N/A

## References


1. Longo DL, Drazen JM. Data Sharing. N Engl J Med. 2016 Jan 21; 374(3): 276–277. doi: 10.1056/NEJMe1516564
2. Greene CS, Garmire LX, Gilbert JA, Ritchie MD, Hunter LE. Celebrating parasites. Nature Genetics. 2017;49(4):483-484. doi:10.1038/ng.3830
3. Byrd JB, Greene CS. Data-sharing models. N Engl J Med 2017; 376:2305-2306. doi: 10.1056/NEJMc1705477
4. Zika virus strain BeH818995 polyprotein gene, complete cds. https://www.ncbi.nlm.nih.gov/nuccore/KU365777.1, accessed 09/25/2018
5. Zika OpenResearch Portal. https://zika.labkey.com/project/OConnor/ZIKV-001/begin.view, accessed 09/25/2018
6. Sudlow C, Gallacher J, Allen N, et al. UK Biobank: An Open Access Resource for Identifying the Causes of a Wide Range of Complex Diseases of Middle and Old Age. PLoS Medicine. 2015;12(3):e1001779. doi:10.1371/journal.pmed.1001779
7. Yenni GM, Christensen EM, Bledsoe EK, Supp SR, Diaz RM, White EP, Ernest SKM. Developing a modern data workflow for evolving data. bioRxiv 344804; 2018; doi:10.1101/344804
8. Alberts B, Kirschner MW, Tilghman S, Varmus H. Rescuing US biomedical research from its systemic flaws. Proceedings of the National Academy of Sciences of the United States of America. 2014;111(16):5773-5777. doi:10.1073/pnas.1404402111
9. Request for Applications: Seed Networks for the Human Cell Atlas. https://www.chanzuckerberg.com/science/rfa/seed-networks, accessed 09/25/2018
10. 2019 Grant Application Guidelines for Young Investigator Grant. https://www.alexslemonade.org/sites/default/files/2019_young_investigator_guidelines_final.pdf, accessed 09/25/2018

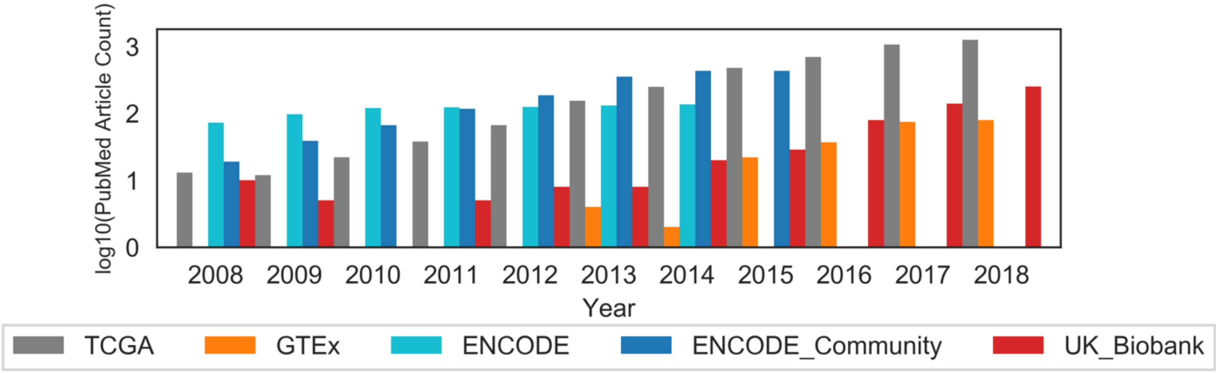


**Figure 1. Citations for publicly shared data between 2008 - present**: For four main public data repositories, the Cancer Genome Atlas (TCGA), Genotype-Tissue Expression Project (GTEx), Encyclopedia of DNA Elements (ENCODE) and UK Biobank, we estimated the number of publications citing each datasets on PubMed (https://www.ncbi.nlm.nih.gov/pubmed/). For ENCODE and ENCODE_Community publications, the ENCODE official website (https://www.encodeproject.org/) was used in place of PubMed for searches up to its finalization in 2016. For the past decade, we estimated that approximately 7,324 publications benefited from these public resources.

Click here to access/download;Figure;Greene Figure 1.png

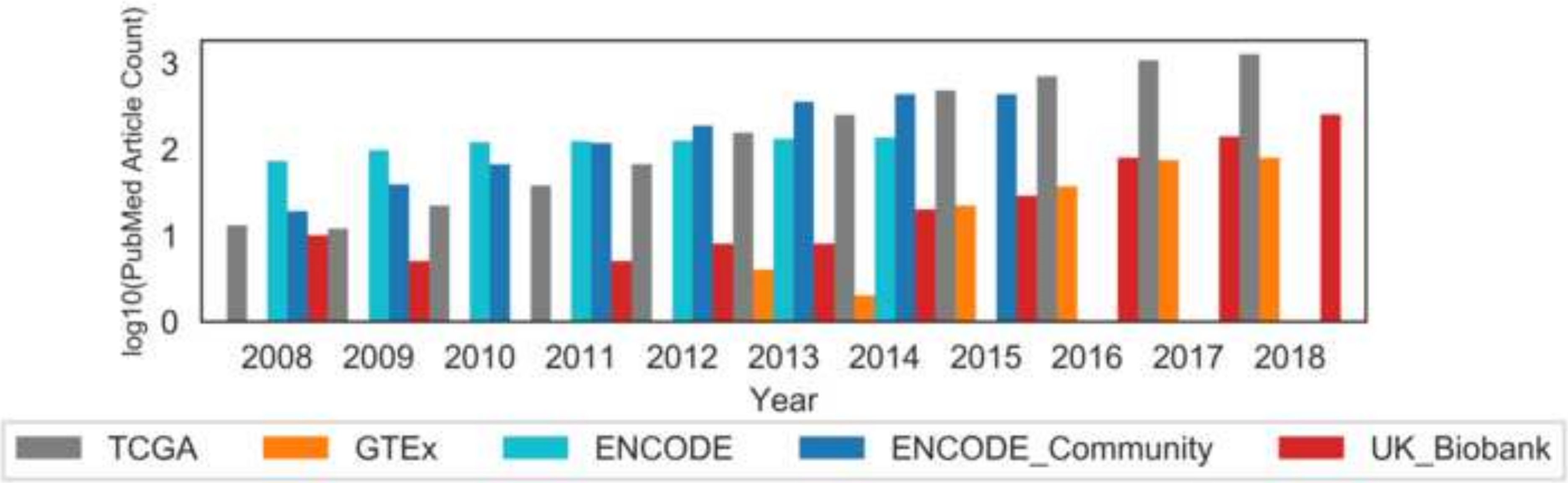

Supplement: GIGA-D-18-00388_Original_Submission.pdf [file giy129_giga-d-18-00388_original_submission.pdf]
